# Severe thoracic trauma caused left pneumonectomy complicated by right traumatic wet lung, reversed by extracorporeal membrane oxygenation support—a case report

**DOI:** 10.1186/s12890-019-0790-1

**Published:** 2019-02-06

**Authors:** Feng Yun Wang, Bin Fang, Zhi Hui Yu, Jing Song Shao, Wei Biao Wen, Li Xin Zhou

**Affiliations:** Critical Care Medicine Department of The First People’s Hospital of Foshan, Lingnan Avenue North 81, Shiwan, Chancheng, Foshan, 528000 China

**Keywords:** Thoracic trauma, Acute respiratory distress syndrome, Extracorporeal membrane oxygenation, Traumatic wet lung, One lung ventilation

## Abstract

**Background:**

Double lumen intubation and one-lung ventilation should be applied without delay in cases of traumatic main bronchial rupture. In most cases, when the patients’ vital signs have been stabilized, the repair can be performed. However, when one-lung ventilation is complicated by traumatic wet lung, the mortality rate is likely to be much higher.

**Case presentation:**

In this case, the patient experienced a left main bronchial rupture, bilateral traumatic wet lung, and acute respiratory distress syndrome (ARDS) because of severe thoracic trauma. Though the patient was treated with intubation and mechanical ventilation (MV), his oxygenation was still not stable. Thus, veno-venous extracorporeal membrane oxygenation (V-V ECMO) was initiated; upon improvement of oxygenation, the patient received an exploratory thoracotomy. Unfortunately, the rupture proved to be irreparable, resulting in a total left pneumonectomy. As there was severe ARDS caused by trauma, ECMO and ultra-low tidal volume (V_T_) MV strategy (3 ml/kg) were utilized for lung protection post-op. ECMO was sustained up to the 10th day, and MV until the 20th day, post-operation. With the support of MV, ECMO and other comprehensive measures, the patient made a recovery.

**Conclusion:**

V-V ECMO and ultra-low V_T_ MV helped this thoracic trauma patient survive the lung edema period and prevented ventilator associated pneumonia (VAP). In extreme situations, with the support of ECMO, the tidal volume may be lowered to 3 ml/kg.

## Background

Blunt traumatic thoracic injuries are often seen in traffic accidents. Unlike usual injuries such as lung contusions, pneumothorax and rib fractures, tracheal ruptures or main bronchial ruptures are uncommon [1]. Taken together the analysis of clinical manifestation, bronchoscopy and computed tomography (CT) scan, the diagnosis usually isn’t difficult to make. After double-lumen intubation and one-lung ventilation (OLV), in the absence of any surgical contraindication, the repair can be performed in most cases [2, 3]. Severe lung contusions or traumatic wet lung are the common causes of acute respiratory distress syndrome (ARDS) [4]. The patient had severe ARDS after severe thoracic trauma, the exaggerated response of innate immunity and the amplification of inflammation were important etiologies for ARDS in the early phase. Research has indicated that the mortality of patients with traumatic lung injuries requiring pneumonectomy is as high as 70%—100% [5, 6]. In the case here addressed, the left main bronchial rupture was irreparable; consequently, the patient had a total left pneumonectomy. The medical focus of this case is OLV complicated by traumatic wet lung, which made the treatment more intricate and decreased the chances of success. However, with the support of mechanical ventilation, extracorporeal membrane oxygenation (ECMO) and other comprehensive measures, the patient survived the right-side traumatic wet lung and ARDS after the left pneumonectomy.

## Case presentation

A 47-year-old male patient, who has no specific past medical history, suffered severe thoracic trauma in a forklift accident 14 h before he was transferred to our hospital. After having his chest crushed by a forklift, the patient instantly had hemoptysis and showed serious signs of respiratory distress. At the local hospital, the physical examination revealed pulse oxygen was at approximately 80%; there was subcutaneous emphysema in the neck and chest; breathing was inaudible by auscultation in the left lung; and, there were moist rales in the right lung. The patient immediately received single-lumen intubation and mechanical ventilation (MV). The CT scan showed left-side pneumothorax, right-side pneumo-hemothorax, bilateral traumatic wet lung, and multiple rib fractures. The bronchoscopy also indicated a left main bronchial rupture. Therefore, the patient was treated immediately with bilateral closed thoracic drainage, fluid infusion, and immobilization of the chest wall.

**Fig. 1:**
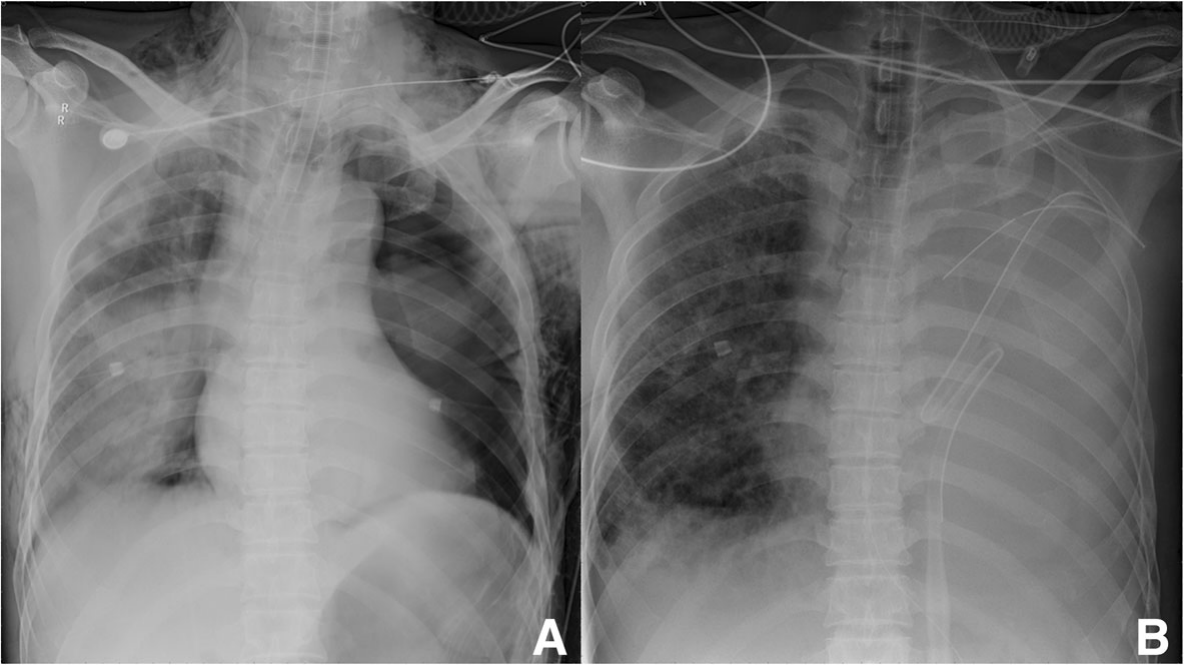
**a** was the x-ray taken on admission, there were bilateral pneumothorax, bilateral traumatic wet lung, and multiple rib fractures. **b** was the x-ray taken after ECMO was weaned, the condition of the right lung recovered significantly, edema and exudation alleviated; the left lung was removed

Treatment notwithstanding, there was no alleviation of the patient’s symptoms, and his pulse oxygen remained consistently low (approximately 80%). Consequently, he was transferred directly to our department. The minute ventilation volume was only 2 to 3  L/min by single-lumen mechanical ventilation. Therefore, the single-lumen tube was replaced with a double-lumen tube, with ventilation only to the right lung to prevent leakage. Nevertheless, the patient’s pulse oxygen remained low, with no remediation of his respiratory distress. On admission, after running the necessary checks and analyses, with his APACHE II score at 25, the predicted odds of mortality was 51%. His blood gas revealed both respiratory acidosis and metabolic acidosis, with both exacerbating gradually. Figure 1 exhibited the chest x-rays at different times, before pneumonectomy (Fig. 1a) and after the withdrawal of ECMO (Fig. 1b).

**Fig. 2:**
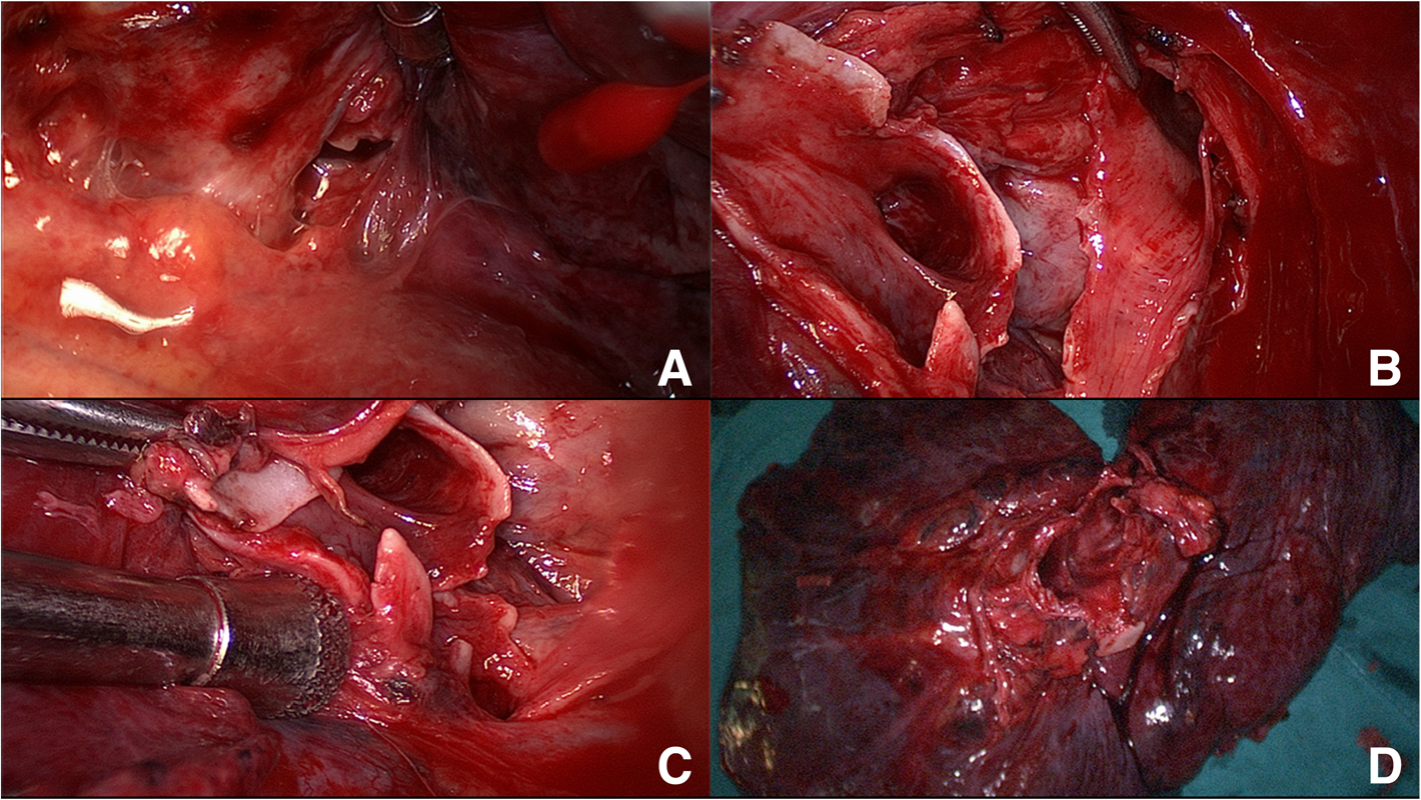
**a**, **b** and **c** were the thoracoscopic pictures taken during the surgery, there were severe edema and congestion in the local tissue; the rupture was shown from different angles, which was irregular and unable to be repaired. **d** was the entire left lung after resection, which was dark red, exhibited serious signs of edema and congestion

At that critical moment, ECMO was initiated without delay. Upon selection of the veno-venous (V-V) ECMO model, catheters were inserted into the right jugular vein (arterial catheter, the tip nearly reached right atrium) and right femoral vein (venous catheter, the tip located at inferior vena cava). Specifically, blood was drawn out from the right atrium to the ECMO device (Maquet, ROTAFLOW Console), after oxygenation it was infused into the right femoral vein, with the gas flow at 4-6 L/min, fraction of inspiration O2(FiO2) at 100% and the pump operating at 3480—3610 rpm. Upon receipt of ECMO and MV, the patient’s oxygenation stabilized; his pulse oxygen rose to 97%—100%; and his respiratory distress was alleviated significantly, thus permitting urgently needed surgery. With the consent of his family members, the patient had an emergency, video-assisted thoracoscopic exploratory thoracotomy. The edema and consolidation of the entire left lung were severe. 1 cm from the tracheal carina, the postero-lateral wall of the left main bronchus experienced an 8 cm long and irregular rupture, which spread to the distal end of the secondary bronchus of the upper and inferior lobes. The rupture was unable to be ordinarily repaired and anastomosed, so the patient required a total left lung resection. The thoracoscopic pictures during the surgery are exhibited in Fig. 2.

After left lung resection, with the support of ECMO, the parameters of ventilator (PURITAN BENNETT 840) were set as follows, mode: Synchronized Intermittent Mandatory Ventilation (SIMV), Frequency(F): 12 times/min, V_T_:200 ml, FiO2: 40%, and positive end expiratory pressure (PEEP): 8cmH2O. Gradually, with ECMO and low tidal volume (V_T_) MV (V_T_ 200 ml) as the main therapy, assisted by anti-inflammatories, antibiotics, sedatives, and analgesics, the patient made a recovery. ECMO was sustained up to the 10th day, and MV until the 20th day, post-operation. After the initiation of ECMO, heparin was micro-pump injected (125u-750u/hour), and activated clotting time (ACT) was monitored every 2 h. ACT was expected to remain between 160 s to 180 s, which was fluctuating between 130 s to 210 s without severe bleeding complication occurring. After the initiation of ECMO, arterial and venous blood gas were tested every 6 h; 24 h before the patient’s ECMO weaning, the gas flow was reduced to 2 L/min; 6 h before weaning to 0 L/min; FiO2 was reduced to 80%, the O2 and CO2 partial pressure of blood gas were dynamically stable, then ECMO was weaned and the related catheters were removed. During the ECMO treatment, infections such as catheter-related bloodstream infection or ventilator associated pneumonia (VAP) should be anticipated, and antibiotics for most gram-negative and some of the sensitive gram-positive bacteria should be applied. In this case, the culture of sputum samples and broncho-alveolar lavage fluid or blood samples were all negative. In the first week after the operation, piperacillin-sulbactam was used to prevent possible lung infections, later to be replaced by imipenem and levofloxacin when the fever and white blood cell count climbed. Ulinastatin, a glycoprotein found in human urine and blood, proved to be a multivalent, Kunitz-type serine protease inhibitor and exhibited moderate anti-inflammatory effects without any immunosuppression side-effects [7]; it was used for immuno-modulation and anti-inflammation in our case. Because the invasive double-lumen intubation and right-side multiple rib fractures caused considerable pain, appropriate analgesics and sedatives were essential for the post-op compliance of the patient. The combination of dexmedetomidine and fentanyl or midazolam and morphine were used alternatively for sedation and analgesia. The alteration reduced the risk of drug accumulation while keeping a satisfying effectiveness. Finally, considering the subcutaneous emphysema in the neck and the edema of bronchial local tissue, a tracheotomy was not performed in the early phase, but a double-lumen tube was retained until the 10th day to cope with possible leakage in the bronchial stump.

## Discussion

This report was about a patient who underwent severe thoracic trauma, resulting in left main bronchial fracture, traumatic wet lung and pneumothorax in both lungs, and multiple rib fractures, the combination of which was very rare, complicated, and fatal. After closed thoracic drainage for both lungs and one-lung ventilation (OLV), the condition of the patient was still aggravating, manifested as severe ARDS, severe acidosis, and hypotension. The deterioration contraindicated anesthesia and decreased the patient’s chances of surviving major surgery—a sure indication for initiating ECMO treatment. The patient was a middle-aged male with no underlying heart disease, and ultrasound showed cardiac function was normal; thus, V-V ECMO was selected. Regularly, after a pneumonectomy, when the function of the contralateral lung is normal, ECMO and MV may be weaned at an early phase. Here, because severe edema and atelectasis of the patient’s right lung caused severe ARDS, lengthened ECMO and MV time were required. With the support of ECMO and MV, the patient survived the pulmonary edema peak period. The lung tissue was able to rest and repair without severe VAP occurring. In view of the severity and complexity of this case, it may guide the treatment of similar cases in the future, especially for thoracic trauma patients with severe ARDS or in need of an extended period of OLV.

Research indicated that even when all conventional treatments failed, ECMO could still improve the survival of thoracic trauma patients [8, 9]. Based on the blood stream access, ECMO is divided into two types: veno-venous and veno-arterial ECMO. The influence of veno-venous ECMO on circulation is slight, for the blood flow is maintained by cardiac function entirely. Therefore, veno-venous ECMO is mainly utilized in cases of non-cardiac acute respiratory failure to improve oxygenation [9–11]. Since veno-arterial ECMO can improve oxygenation, as well as provide cardiac support, it is mainly utilized in severe heart failure or heart transplantation [12]. Along with the progress in lung injury repair, concomitantly, the oxygen partial pressure escalated and the CO2 partial pressure deescalated in both arterial and venous blood gas. In spite of this, for the sake of lung protection, the FiO2 of ECMO was kept between 70 to 100% without further reduction in the treatment. The blood flow was sustained at a constant rate of 4.0–4.5 L/min. As higher FiO_2_ and blood flow representing more oxygenation support, would ensure the ventilator functioning at low parameters to protect the injured lung. In the ECMO treating period, appropriate anti-coagulation measures should be applied to prevent thrombosis from occurring in the device. However, in traumatic and post-operation patients, anti-coagulation might cause severe organ bleeding complications, which should be alerted during the treatment. A comparably higher blood flow means the ECMO would work at a comparably higher pump speed, which would not only reduce the risk of blood clotting in the device but also reduce bleeding complications as less heparin would be needed. In our case, though the trauma was extensive and severe, the high blood flow of ECMO and low dosage of heparin post-op reduced the bleeding risk ideally.

The robust oxygenation support by ECMO not only let us keep V_T_ at 200 ml, F at 12 times/min constantly to decrease the risk of barotrauma and let the lung have sufficient rest, but also allowed the FiO2 of ventilator to be kept constantly at 40%, a relatively low value, to further decrease oxidative stress injury and prevent pulmonary fibrosis. Since OLV and severe acute lung injury simultaneously exist, low tidal volume ventilation is a crucial strategy for lung protection. With the support of ECMO, regardless of the severe lung edema caused by trauma, the oxygen supply was sufficient and CO_2_ could be removed swiftly from the blood. Therefore, V_T_ could be lowered to 3.0 ml/kg, which is beneficial for lung repair, but might increase the odds of atelectasis. Lung recruitment maneuvers and selection of an appropriate PEEP might be suitable measures for coping with this concern. Though V_T_ and F were kept low for lung protection, PEEP was kept at 5–8 cmH_2_O to prevent lung atelectasis before ECMO weaning. With reference to blood gas and chest x-rays, when lung recruitment was deemed necessary, for the sake of lung protection, a respiratory balloon was used manually to expand the lung, no other measures taken in this case. The patient had injured both lungs as well as the pneumothorax in both sides, any lung recruitment measures that may significantly increase airway pressure were not considered. Recently, similar ultra-low tidal volume, protective ventilation strategy and extracorporeal CO_2_ removal membrane were also used successfully to treat near-fatal asthma [13]. In extremely severe cases, in combination with ECMO and MV, prone positioning may provide some extra respiratory support [14, 15]. The patient’s multiple rib fractures were the main reason prevented us from using this therapy; fortunately, after ECMO initiation, the patient’s vital signs stabilized.

## Conclusion

Multi-disciplinary cooperation is needed for the treatment of severe thoracic trauma. A main bronchial rupture may be complicated by contralateral conditions such as traumatic wet lung, which could dramatically increase treatment difficulty. Severe traumatic wet lung may result in ARDS, which may need ECMO for advanced life support when necessary. In the acute lung edema phase, low tidal volume ventilation should be favored to reduce barotrauma as well as to sustain the patient.

## Abbreviations

ARDS: Acute respiratory distress syndrome
CT: Computed tomography
ECMO: Extracorporeal membrane oxygenation
MV: Mechanical ventilation
PEEP: Positive end expiratory pressure
VAP: Ventilator Associated Pneumonia
